# Virulence, antimicrobial resistance, and molecular characteristics of carbapenem-resistant *Klebsiella pneumoniae* in a hospital in Shijiazhuang City from China

**DOI:** 10.1007/s10123-023-00357-x

**Published:** 2023-04-25

**Authors:** Yumei Guo, Faqiang Liu, Yulan Zhang, Xiaoli Wang, Weili Gao, Baohong Xu, Yuxue Li, Ning Song

**Affiliations:** 1 Hebei Provincial Key Research Laboratory of Intractable Bacteria, Shijiazhuang Center for Disease Control and Prevention, Shijiazhuang, China; 2 Department of Infectious Diseases, The Second Hospital of Hebei Medical University, Shijiazhuang, China; 3 Department of Clinical Laboratory, Shijiazhuang People’s Hospital, Shijiazhuang, China

**Keywords:** *Klebsiella pneumoniae*, Carbapenem resistance, Antimicrobial resistance, Pulsed-field gel electrophoresis (PFGE), Hypervirulent *Klebsiella pneumoniae* (HVKP)

## Abstract

Carbapenem-resistant *Klebsiella pneumoniae* (CRKP), as one of the most common drug-resistant bacteria threatening human health, is hyper-resistant to multiple antimicrobial drugs and carbapenems, which can be dealt with only limited clinical treatment options. This study described the epidemiological characteristics of CRKP in this tertiary care hospital from 2016 to 2020. Specimen sources included blood, sputum, alveolar lavage fluid, puncture fluid, secretions from a burn wound, and urine. Among the 87 carbapenem-resistant strains, ST11 was the predominant isolate, followed by ST15, ST273, ST340, and ST626. These STs were in broad agreement with the STs defined by pulsed-field gel electrophoresis clustering analysis in discriminating clusters of related strains. Most CRKP isolates contained the *bla*KPC-2 gene, some isolates carried the *bla*OXA-1, *bla*NDM-1, and *bla*NDM-5 genes, and the isolates carrying carbapenem resistance genes were more resistant to the antimicrobials of β-lactams, carbapenems, macrolides, and fluoroquinolone. The OmpK35 and OmpK37 genes were detected in all CRKP strains, and the Ompk36 gene was detected in some CRKP strains. All detected OmpK37 had 4 mutant sites, and OmpK36 had 11 mutant sites, while no mutant sites were found in OmpK35. More than half of the CRKP strains contained the OqxA and OqxB efflux pump genes. The virulence genes were most commonly combined with urea-wabG-fimH-entB-ybtS-uge-ycf. Only one CRKP isolate was detected with the K54 podoconjugate serotype. This study elucidated the clinical epidemiological features and molecular typing of CRKP, and grasped the distribution of drug-resistant genotypes, podocyte serotypes, and virulence genes of CRKP, providing some guidance for the subsequent treatment of CRKP infection.

**Supplementary Information:**

The online version contains supplementary material available at 10.1007/s10123-023-00357-x.

## Introduction

*Klebsiella pneumoniae* is a gram-negative bacterium that is widely distributed in the environment such as surface water, sewage, soil, and plants, and it may also exist in the intestinal tract and nasopharynx of humans (Podschun and Ullmann 1998). CRKP is an increasingly important pathogen of hospital-acquired infections that may cause a wide range of infections, including urinary tract infections, pneumonia, bacteremia, liver abscesses, and brain abscesses, and may be life-threatening in severe infections (Paczosa and Mecsas 2016). In clinical practice, great success has been achieved in the treatment of pathogenic infections with antimicrobials, but in recent years, *K. pneumoniae* has rapidly developed resistance to all known antimicrobials, including carbapenems, and this phenomenon can be seen all over the world. Due to its highly transmissible nature, the emergence of carbapenem-resistant *K. pneumoniae* in endemic areas of *Klebsiella pneumoniae* may lead to a dangerous local increase in the number of carbapenem-resistant *K. pneumoniae*, and the emergence of carbapenem-resistant *K. pneumoniae* has clearly become a world public health problem that may be faced by all the human beings (Holden et al. 2018; Doi 2019; Karampatakis et al. 2016). The main resistance mechanisms of carbapenem-resistant *K. pneumoniae* are currently shown as follows: (1) *K. pneumoniae* can be prevented by encoding genes (e.g., New Delhi metallo-β-lactamase [NDM], *K. pneumoniae* carbapenemase [KPC], Verona integron-encoded metallo-β-lactamase [VIM], oxacillin-hydrolyzing β-lactamase [OXA], and imipenem-hydrolyzing β-lactamase [IMP]) to hydrolyze carbapenemases produced by the carbapenemase, which is an enzyme with a positive effect on the hydrolysis of carbapenems. Carbapenemases are catalytically efficient endocannabinoid enzymes that may result in elevated minimum inhibitory concentrations of carbapenem and include classes A, B, and D. The three major families of class-A serine carbapenemases include NMC/IMI, SME, and KPC enzymes; the most common families of class-B metallo-lactamases include *bla*NDM, *bla*VIM, *bla*IMP, *bla*GIM, and *bla*SIM enzymes; class-D serine carbapenemase includes *bla*OXA β-lactamase. (2) The expression of efflux pump gene allows bacterial cells to actively and rapidly squeeze out carbapenems, avoiding the bacterial cell wall from being damaged by carbapenems. (3) The permeability of the outer membrane is reduced through the production of β-lactamases (AmpC) and the alterations in the bacterial cell membrane (pore protein mutations in OmpK35 and OmpK36), thus blocking carbapenem drugs from entering into the bacteria (Alizadeh et al. 2018; Durante-Mangoni et al. 2019; Girlich et al. 2019; Queenan and Bush 2007). The synthesis of carbapenemase-encoding genes has caused numerous difficulties for clinicians, and the drugs do not work as well as they should, making it difficult to develop treatment plans for patients. The common carbapenemase-encoding genes in China are mainly KPC, NDM, and IMP n (Zhang et al. 2020a). A weak correlation exists between the expression of carbapenem antimicrobial resistance in CRKP and the virulence genes they possess, and these strains may carry multiple virulence genes that may have the ability to encode capsules (magA, wcaG), express high viscosity, mucus phenotype regulators (magA, rpmA), produce mycobacterial hair adhesion elements (fimH), synthesize lipopolysaccharides (wabG, uge, ycf), and construct iron acquisition systems (iutA, iroN, entB, KfuB), iron carriers (aerobactin), associated with allantoin metabolism (allS), yersin bacteriocin (ybtS), and other virulence factors (vatD); the functions possessed by these virulence genes allow them to overcome host defenses (Hartman et al. 2009; Yu et al. 2008, 2006, 2007; Remya et al. 2019; Candan and Aksöz, 2015). Almost all CRKPs prevalent worldwide are multi-locus sequence typing (ST) 258 and its related variants; for example, ST11, which is widely prevalent in China, differs from ST258 in only one gene locus, but it is not yet clear why ST258 and its related variants spread so widely and successfully (Qi et al. 2011; Chen et al. 2014).

In this study, the antimicrobial susceptibility test was performed on 87 carbapenem-resistant *K. pneumoniae* strains. Multi-locus sequence typing, drug resistance genes, virulence genes, and capsular serotypes were detected using PCR amplification; clonal relationship detection was performed using pulsed-field gel electrophoresis (PFGE) technique to clarify the molecular epidemiological characteristics of CRKP in this hospital.

## Materials and methods

### Sample collection and bacterial isolates

A total of 87 non-replicated carbapenem-insensitive *K. pneumoniae* strains were collected from January 2016 to December 2020 in a tertiary care hospital in Shijiazhuang, Hebei Province, China. All isolates of specimens (sputum, secretions, urine, alveolar lavage, puncture fluid, and blood) were obtained from the clinical departments of this tertiary hospital, and there was no restriction on age, sex, disease type, and clinical department of the patients who provided the specimens. Eighty-seven isolates were identified using a MALDI-TOF mass spectrometer (AUTOFLEXSpeed, Germany). The MIC values of imipenem (MIC ≥ 4 μg/mL), meropenem (MIC ≥ 4 μg/mL), and ertapenem (MIC ≥ 2 μg/mL) were determined using the microbroth dilution method with a fold reference (CLSI M100, 2021). According to the guidelines in the Clinical and Laboratory Standards Institute (CLSI) (2019, 29), the inclusion criteria for the guidelines were reduced susceptibility to at least one carbapenem (imipenem, meropenem, and ertapenem) as carbapenem resistance. All 87 isolates were stored in a − 80℃ refrigerator.

### Antimicrobial sensitivity experiments

Antimicrobial susceptibility testing is conducted using the microdilution method (MIC method) according to the manufacturer ‘s instructions. To put it simply, a fresh bacterial strain is prepared into a 0.5 mcf suspension, added to a 96-well plate following the instructions, and incubated in a constant temperature incubator for 24 h. The number of negative wells for each antimicrobial is then recorded and entered into the result analysis system provided by the manufacturer to obtain the corresponding antimicrobial ‘s minimum inhibitory concentration. Finally, these figures are entered into the manufacturer ‘s result analysis system to obtain the minimum inhibitory concentration. Thirty-one antimicrobials used in antimicrobial trials included ampicillin, ampicillin-sulbactam, tetracycline, chloramphenicol, trimethoprim-sulfamethoxazole, cefazolin, cefotaxime, ceftazidime, cefoxitin, gentamicin, imipenem, Nalidixan, azithromycin, sulfisoxazole, ciprofloxacin, amoxicillin-clavulanate, cefotaxime-clavulanate, ceftazidime-clavulanate, colistin, polymyxin B, minocycline, amikacin, aztreonam, cefepime, meropenem, ertapenem levofloxacin, doxycycline, kanamycin, streptomycin, and gemifloxacin. The breakpoint of measured antimicrobial MIC values was obtained according to the corresponding standards of the American Committee for Clinical Laboratory Standardization (CLSI M100, 2021) for the corresponding sensitive (S), moderately sensitive (I), and resistant (R) results. The antimicrobial sensitivity test was performed using *E.coli* ATCC25922 and *K. pneumoniae* ATCC700603 as the reference standard.

### Drug resistance gene, capsular serotype, and virulence gene detection

The polymerase chain reaction was performed using specific primer sequences with TaKaRa ‘s Premix Taq configuration system using PTC-200 and Veriti. Twelve resistance genes (*bla*KPC, *bla*NDM, *bla*VIM, *bla*OXA-48, *bla*OXA-1-like, blaIMP, *bla*GES, *bla*SHV, *bla*CTX-M-65, *ompK35*, *ompK36*, *ompK37*), 16 common virulence genes (*WcaG*, *ureA*, *wabG*, *fimH*, *entB*, *iutA*, *ybtS*, *rmpA*, *IroN*, *KfuB*, *aerobactin*, *alls*, *uge*, *vatD*, *magA*, *and ycf)*, and seven capsular serotype genes (K1, K2, K3, K5, K20, K54, and K57) were selected (Kaczmarek et al. 2006; Dallenne et al. 2010; Zhan et al. 2017; Zhang et al. 2018). The reaction program for the total reaction system of 25 μL was set as follows: pre-denaturation temperature 94 °C for 2 min, denaturation temperature 94 °C for 20 s, annealing temperature depending on different primers (*bla*KPC, *bla*VIM, *bla*IMP, *ompK35*, *ompK36*, and *ompK37* 55 °C; *bla*GES and *bla*OXA-48 57 °C; *bla*OXA-1-like and uge 60 °C; *bla*SHV, K20, entB, and *ybtS* 56 °C; *bla*CTX-M-65, *bla*NDM, K1, and *WcaG* 54 °C; *ureA*, *wabG*, *ycf*, *rmpA*, *aerobactin*, *IroN*, *KfuB*, *alls*, K3, K5, K54, and K57 58 °C; K2 64 °C; *fimH* 62 °C; *iutA* 66 °C; *vatD* and *magA* 48 °C) (Kaczmarek et al. 2006; Dallenne et al. 2010; Zhan et al. 2017; Zhang et al. 2018), time 30 s, extension temperature 72℃, number of amplification cycles 35, final extension temperature 72℃, time 5 min. PCR products were analyzed using a fully automated electrophoresis analyzer (QIAXEL) and Sanger sequencing was performed (sent to Beijing DynaBio), and the sequencing results were analyzed using NCBI BLAST (https://blast.ncbi.nlm.nih.gov/blast.cgi) for comparative analysis.

### Detection of efflux pump gene

7500 Real-Time PCR System (Applied Biosystems) was used to detect OqxA and OqxB efflux pump genes, primers were synthesized by Beijing DynaBio, the primer system used was TaKaRa ‘s Premix Ex TaqTM (Probe qPCR), and the amplification procedure was as follows: initial incubation at 95 °C for 2 min, followed by 40 cycles of 10 s at 95 °C, 30 s at 60 °C, and 10 s at 72 °C (Zhong et al. 2014). The strains that tested positive for DNA were extracted and reverse-transcribed into cDNA using TaKaRa ‘s PrimeScript™ II 1st Strand cDNA Synthesis Kit s. The strains were then configured using the Premix Ex Taq™ system and subjected to real-time fluorescent quantitative PCR. Amplification conditions are as described above. The 2^-ΔΔCT of each strain was calculated as the relative expression of the efflux pump gene for each strain, using the rpoB gene as the reference gene and strain number 20021762; the CRKP strain as the reference. Each sample was processed in triplicate.

### Multi-locus sequence typing (MLST)

PCR amplification of seven housekeeping genes of *K. pneumoniae* (*gapA*, *infB*, *mdh*, *pgi*, *phoE*, *rpoB*, *and tonB*) was also performed using TaKaRa ‘s Premix Taq configuration system, polymerase chain reaction using PTC-200 and Veriti, and total reaction system 50 μL. The reaction program was set as follows: pre-denaturation at 94 °C for 2 min, denaturation at 94 °C for 20 s, annealing temperature depending on different primers (*gapA* 60 °C, *TonB* 45 °C, *infB*, *mdh*, *pgi*, *phoE*, and *rpoB* all 50 °C) for 30 s, extension at 72 °C for 30 s, number of amplification cycles 35, final extension at 72 °C for 5 min. The PCR products were analyzed using a fully automated electrophoresis analyzer (QIAXEL), and Sanger sequencing was performed (sent to Beijing DynaScience Biotech). The sequencing results were typed for STs using the BIGSdb-Pasteur database (https://bigsdb.pasteur.fr/klebsiella/klebsiella.html).

### Pulsed-field gel electrophoresis (PFGE)

PFGE assays were performed on all collected carbapenem-resistant *K. pneumoniae* strains. Briefly, the genomic DNA of carbapenem-resistant *K. pneumoniae* was prepared by embedding *K. pneumoniae* cells in Seakem Gold Agarose and then digesting them using *XbaI* enzymes at 37 °C for 4 h. The genomic DNA of carbapenem-resistant *K. pneumoniae* was then stained using a Bio-Rad CHEF III system (120° angle, 6 V/cm, switching time 6 s, 36 s) at 14 °C for 18.5 h. After completion of electrophoresis, DNA bands were stained using GelRed and imaged with a gel imaging system (BIO-RAD Molecular Imager® Gel DocTM XR + with Image LabTM software). Salmonella serovar Brendaup strain H9812 was used as a molecular marker. DNA profiles were interpreted using the criteria of Tenover et al. (1995) (Zhan et al. 2017). PFGE patterns were analyzed using Bionumerics software (Applied Mathematics, Sint-Martens-Latem, Belgium) using dice similarity coefficients. The isolates with a similarity of more than 80% were defined as identical PFGE clusters.

## Results

### Sample types and sources of strains isolated from K. pneumonia

The number of CRKPs isolated per year from 2016 to 2020 in this tertiary care hospital was 14, 5, 6, 28, and 34 strains, respectively. The vast majority of the isolated CRKP specimens were originated from sputum 78.16% (68/87), followed by burn secretions 9.20% (8/87), urine 3.45% (3/87), alveolar lavage fluid 3.45% (3/87), puncture fluid 3.45% (3/87), and blood 2.30% (2/87). The detection rate of CRKP in the lower respiratory tract was high; CRKP was also widely distributed in clinical departments of hospitals, with the majority of carbapenem-resistant *K. pneumoniae* coming from the respiratory medicine department 21.84% (19/87), intensive care unit 21.84% (19/87), burns department 16.09% (14/87), rehabilitation department 13.79% (12/87), geriatrics department 9.20% (8/87), neurosurgery department 4.60% (4/87), thoracic surgery department 2.30% (2/87), nephrology department 2.30% (2/87), hematology department 2.30% (2/87), general surgery department 2.30% (2/87), neurology department 1.15% (1/87), gastroenterology department 1.15% (1/87), and endocrinology department 1.15% (1/87). Respiratory and Critical Care Medicine remain the main clinical departments prevalent with carbapenem-resistant *K. pneumoniae*. Although there are relatively fewer carbapenem-resistant *K. pneumoniae* in other departments, there is a small amount of CRKP epidemic transmission.

### Antibacterial drugs, carbapenem-resistant genes, and efflux pump genes

**Fig. 1:**
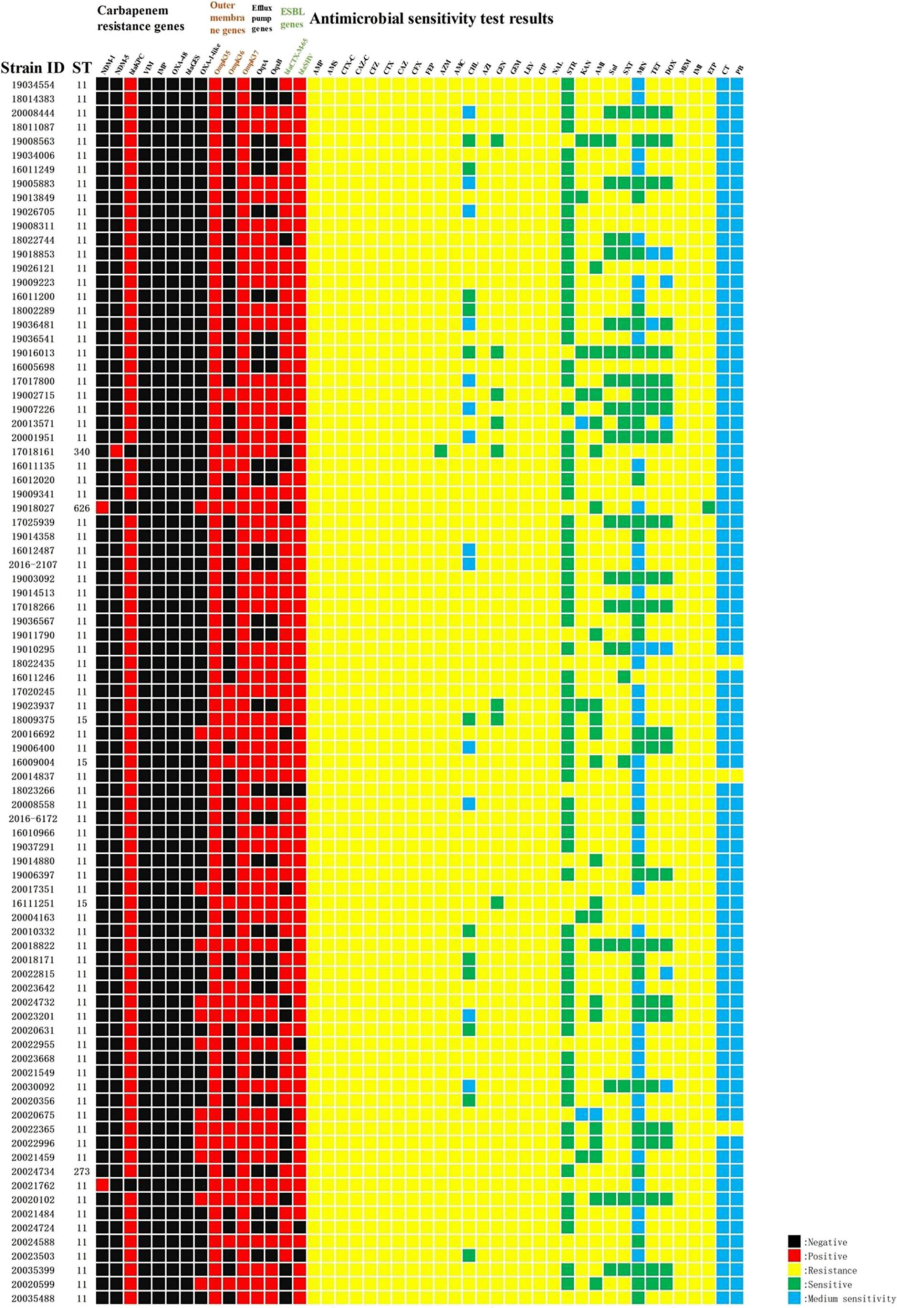
The test results of ST type, carbapenem resistance genes, outer membrane genes, efflux pump genes, and antimicrobial susceptibility for 87 CRKP strains

**Fig. 2:**
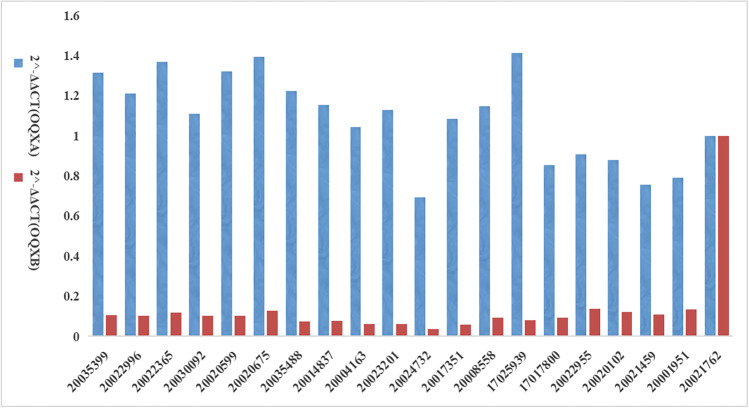
Relative expression of 19 strains of KPC-2 CRKP efflux pump genes. Note: the blue bars represent OqxA gene test results and the brown bars represent OqxB gene test results. The vertical coordinate represents the 2^-ΔΔCT of the oqxA/B gene and the horizontal coordinate represents the strain number

Thirty-one antimicrobials commonly used in clinical practice were selected according to the microbroth dilution method (MIC method), which was developed according to the manufacturer ‘s method. Ampicillin, ampicillin-sulbactam, ceftazidime clavulanic acid, ceftazidime clavulanic acid, cefazolin, ceftazidime, cefoxitin, cefepime, amoxicillin-clavulanic acid, gemifioxacin, levofloxacin, ciprofloxacin, nalidixic acid, meropenem, imipenem, and azithromycin are antimicrobial drugs with a 100% resistance rate. The resistance rate is 98.85% for amineptide and ertapenem. The resistance rates for gentamicin, kanamycin, sulfisoxazole, trimethoprim, amikacin, chloramphenicol, tetracycline, doxycycline, streptomycin, and minocycline were 90.80%, 89.66%, 79.31%, 77.01%, 72.41%, 71.59%, 70.11%, 66.67%, 21.84%, and 10.31%. Colistin and polymyxin B’s resistance rate is 3.45%. The specific MIC values of the 31 antimicrobials are shown in the attached information. The resistance rate of CRKP to 20 antimicrobials in 31 antimicrobial sensitivity assays exceeded 90%, with an alarming 100% resistance rate of CRKP to 17 antimicrobials. All CRKPs were multi-drug-resistant (MDR), with one strain being insensitive to β-lactams, chloramphenicol, macrolides, fluoroquinolones, aminoglycosides, sulfonamides, tetracyclines, carbapenems, and glycopolypeptide antimicrobials. The distribution of resistant genotypes among 87 carbapenem-resistant *K. pneumoniae* strains was *bla*KPC-2 type 96.55% (84/87), *bla*OXA-1 family group 14.94% (13/87), *bla*NDM-1 type 2.30% (2/87), and *bla*NDM-5 type 1.15% (1/87), while *bla*VIM, *bla*IMP, and *bla*OXA-48 resistance genes were not detected; the extended-spectrum-beta-lactamase genes *bla*SHV 95.40% (83/87), *bla*CTX-M-65 75.86% (66/87); the outer membrane genes OmpK35 100% (87/87), OmpK36 22.99% (20/87), and OmpK37 100% (87/87); and the efflux pump genes OqxA 63.22% (55/87) and OqxB 63.22% (55/87). The detailed distribution of drug resistance genes is shown in Table 1. No mutation sites were found in all detected CRKPs of OmpK35, while all detected OmpK37 genes had four mutation types of p.I70M, p.I128M, m233_None234insQ, and p.N230G. The GenBank ID of the pore protein sequence of the reference *Klebsiella pneumoniae* strain of OmpK37 was AJ011502. Eleven types of mutation were also detected in the OmpK36 gene, including p.F207W, p.D224E, p.L59V, p.L228V, p.N49S, p.Q227_None679del, p.L191S, p.A190_None568del, p.N218H, p. E232R, and p.A217S. The GenBank accession number of the pore protein sequence of the *Klebsiella pneumoniae* strain referenced by OmpK36 was Z33506.1. Detailed results of carbapenem resistance genes, outer membrane protein genes, efflux pump genes, extended-spectrum-beta-lactamase genes, and antimicrobial sensitivity test of each isolated strain are shown in Fig. 1. We selected 19 CRKP strains of KPC-2 type for mRNA expression assay of exocytosis pump gene. Expression of mRNA for the efflux pump gene was detected in all 19 CRKP strains. The relative expression of the efflux pump genes is detailed in Fig. 2.

**Table 1:** Distribution of drug resistance genes and virulence genes of 87 isolates

Drug resistance gene and virulence genes	Quantity	Total volume	Results
Carbapenem resistance genes			
KPC-2	84	87	96.55%
OXA-1-like	13	87	14.94%
NDM-1	2	87	2.30%
NDM-5	1	87	1.15%
GES	0	87	0.00%
OXA-48	0	87	0.00%
VIM	0	87	0.00%
IMP	0	87	0.00%
Outer membrane genes			
OmpK35	87	87	100.00%
OmpK37	87	87	100.00%
OmpK36	20	87	22.99%
Efflux pump genes			
OqxA	55	87	63.22%
OqxB	55	87	63.22%
Extended-spectrum-beta-lactamase genes			
SHV	83	87	95.40%
CTX-M-65	66	87	75.86%
Virulence genes			
WabG	87	87	100.00%
entB	87	87	100.00%
ycf	87	87	100.00%
ureA	82	87	94.25%
ybtS	80	87	91.95%
uge	77	87	88.51%
fimH	73	87	83.91%
iutA	33	87	37.93%
rmpA	23	87	26.44%
WcaG	9	87	10.34%
magA	3	87	3.45%
KfuB	1	87	1.15%
alls	1	87	1.15%
iroN	0	87	0.00%
aerobactin	0	87	0.00%
vatD	0	87	0.00%

### Capsular serotypes and virulence genes

**Fig. 3:**
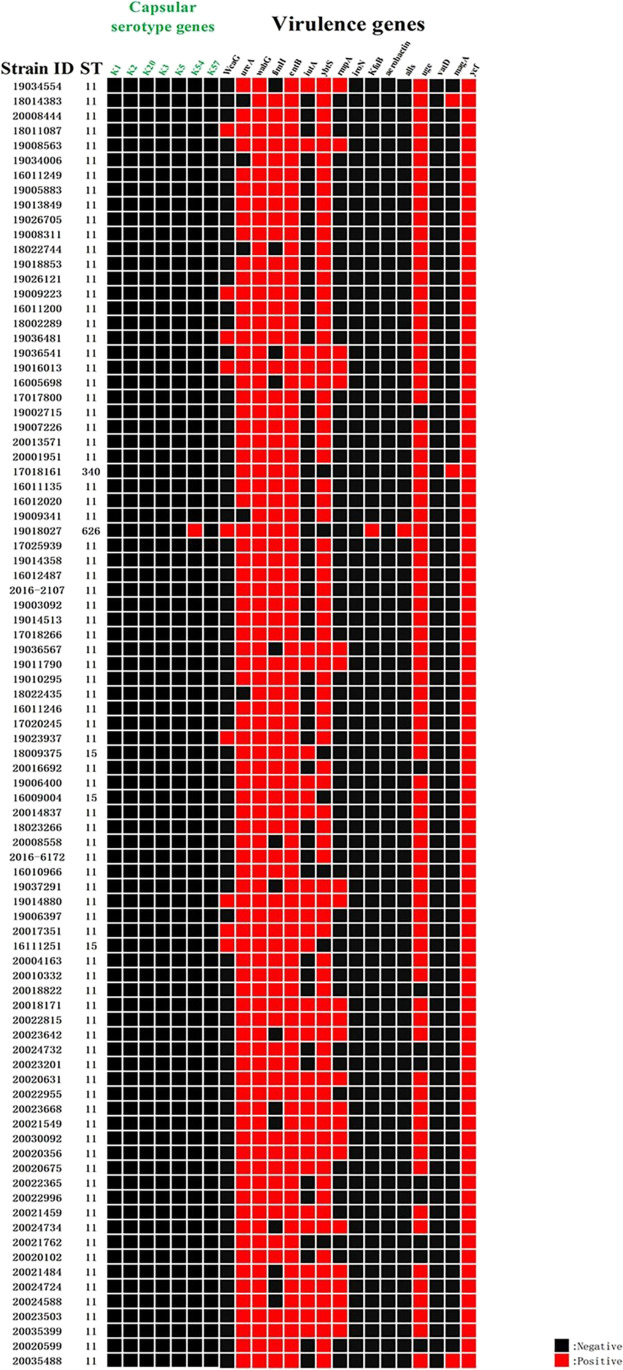
ST type, capsular serotype, and virulence gene detection results of 87 CRKP strains

The results were verified by amplification of specific primers, the amplified products were verified by electrophoresis, and the products verified by electrophoresis were sent to the company for sequencing, and the sequencing results were verified by BLAST. Only K54 1.15% (1/87) of the 87 isolated carbapenem-resistant *K. pneumoniae* were detected in the capsular serotype, whereas K1, K2, K3, K5, K20, and K57 capsular serotypes were not detected. Virulence genes were detected in 100.00% (87/87) of *WabG*, 100.00% (87/87) of *ycf*, 100.00% (87/87) of *entB*, 94.25% (82/87) of *urea*, 91.95% (80/87) of *ybtS*, 88.51% (77/87) of *uge*, and 83.91% (73/87) of *fimH*. Detailed distribution of virulence genes is shown in Table 1. The most common combination of virulence genes in this study was *urea-wabG-fimH-entB-ybtS-uge-ycf*; the virulence gene combination in the CRKP strain with capsular serotype K54 was *WcaG-urea-wabG-fimH-entB-KfuB-alls-uge-ycf*. The detailed results are shown in Fig. 3.

### Molecular characteristics of K. pneumoniae isolates

**Fig. 4:**
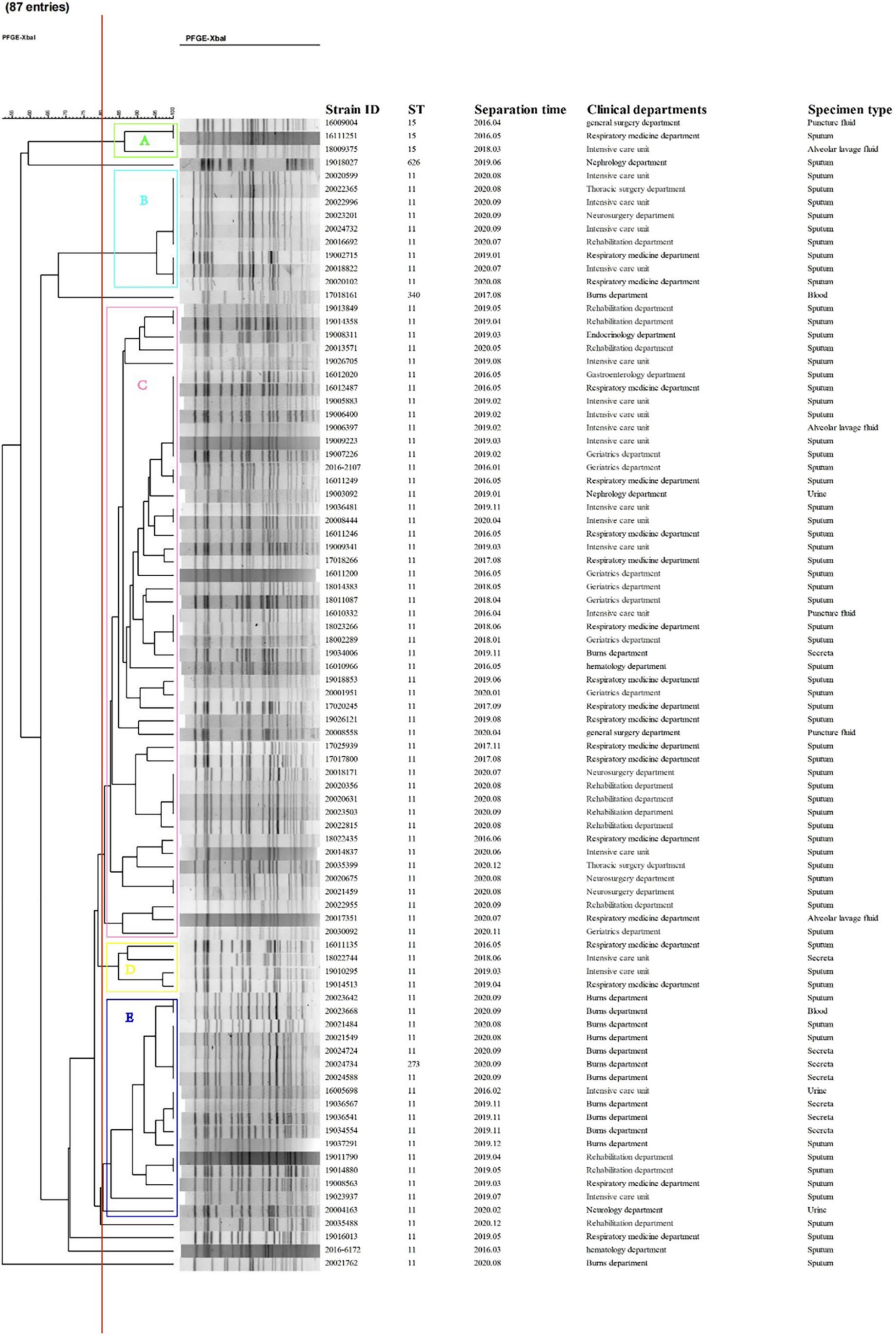
Pulse-field gel electrophoresis results of 87 strains of CRKP. Note: the red line is the dividing line with 80% similarity, the light green box represents cluster A; the sky blue box represents cluster B; the pink box represents cluster C; the light yellow box represents cluster D; the dark blue box represents the E cluster

Among the 87 CRKP strains, five STs were identified, including ST11 93.10% (81/87), ST15 3.45% (3/87), ST273 1.15% (1/87), ST340 1.15% (1/87), and ST626 1.15% (1/87); the results of PFGE clustering analysis showed that 87 CRKP strains could be divided into five clusters: cluster A comprised of ST15 3.45% (3/87), cluster B comprised of ST11 10.34% (9/87), cluster C comprised of ST11 55.17% (48/87), cluster D comprised of ST11 4.60% (4/87), cluster E comprised of ST11 18.39% (16/87), and ST273 1.15% (1/87). The other six CRKP strains were not clustered with other strains, including ST11 4.60% (4/87), ST626 1.15% (1/87), and ST340 1.15% (1/87). The detailed results are shown in Fig. 4.

## Discussion

The globally first case of *K. pneumoniae* type *bla*KPC was discovered in the USA in 1996. Over time, carbapenem-resistant bacteria capable of producing *bla*KPC have spread to most areas in the USA, Israel, and southern European countries (especially Greece and Italy), as well as to the South American continent and China (Munoz-Price et al. 2013). The first *bla*KPC-2 strain of *K. pneumoniae* was discovered in Zhejiang Province of China in 2007, and since then *bla*KPC-2 pneumoniae has spread rapidly to other provinces and cities such as Jiangsu (Qi et al. 2011), Anhui (Qi et al. 2011), Fujian (Chen et al. 2020), Shanghai (Tian et al. 2018), Chongqing (Huang et al. 2021), and Beijing(Yan et al. 2019). Some studies suggested that the plasmids encoding the KPC-2 gene were different in different regions and that the propagation of *bla*KPC in these CRKPs was due to the horizontal transfer capacity of the plasmids or the mobilization of genetic elements in the plasmids, such as transposons and insertion sequences (Qi et al. 2011; Cuzon et al. 2010). In this study, we described the molecular epidemiological trends of CRKP in a tertiary hospital in China from 2016 to 2020, where the incidence of CRKP infections increased during this 5-year period and the overall number of CRKP detections showed a dramatic annual increase. The results highlighted the ability of horizontal transfer of the carbapenemase gene of CRKP to cooperate with clonal transmission, leading to the rapid spread of CRKP within this tertiary care hospital (Cuzon et al. 2010; Han et al. 2020). Type *bla*KPC-2 of *K. pneumoniae* was also the main prevalent carbapenemase type isolated from this tertiary care hospital, which was consistent with the molecular epidemiological findings of CRKP conducted in most regions of China (Wang et al. 2018). Among the strains collected, however, we found two carbapenemase types *bla*NDM-1 and *bla*NDM-5, which were rare in tertiary care hospitals. And some studies suggested that *bla*NDM-1 CRKP was more likely to cause infection in children (Qamar et al. 2019), *bla*NDM-1 and *bla*NDM-5 were considered to be more resistant than *bla*KPC-2 in previous studies, but in this study, *bla*NDM-1 and *bla*NDM-5 did not show any significant difference in resistance compared with *bla*KPC-2. And even more so, a CRKP strain of *bla*KPC-2 was not sensitive to any of the 30 antimicrobials applied in this study, which seemed to imply that the resistance of *bla*KPC to antimicrobials in this hospital was gradually evolving (Walsh et al. 2011; Nakano et al. 2014; Jin et al. 2021). The outer membrane protein gene is also an important factor in the regulation of resistance in CRKP strains, and in our study, we did not find strains with simultaneous deletion of OmpK35, Ompk36, and OmpK37, which suggested that clinical strains of *Klebsiella pneumoniae* with simultaneous deletion of the pore protein gene were uncommon (Doménech-Sánchez et al. 2000). OmpK35 and OmpK37 were detected in all CRKP isolates, and OmpK36 was detected in some CRKP isolates. Some researchers showed that OmpK37 was preferentially expressed in the strains with outer membrane protein deletions, and all the OmpK37 we detected had four mutant loci and OmpK36 had eleven mutant loci, suggesting that the mutant loci in the strains may be related to their resistance to antibiotics, as they may affect the function and structure of bacterial membrane proteins and thus increase the stability of the strains. Therefore, the detection of identical mutant sites in different strains may indicate that these mutations are highly beneficial for the survival and reproduction of the strain or are the product of special adaptation to certain environmental stresses (Kaczmarek et al. 2006; Doménech-Sánchez et al. 1999). It was also shown that the strains with simultaneous deletion of OmpK35 and OmpK36 tended to be more resistant, but the deletion of a single pore protein gene also played a role in the enhancement of bacterial resistance (Shakib et al. 2012; Hernández-Allés et al. 1999). In our study, the relative expression of the oqxA gene in the 19 CRKP strains sampled did not differ significantly from that of 20,021,762 while the relative expression of the oqxB gene was less than that of 20,021,762. The OqxAB efflux pump gene was detected in more than half of our CRKP isolates, and OqxAB contributed significantly to the development of resistance to the antimicrobials of quinolones, tetracyclines, and chloramphenicol in *Klebsiella pneumoniae* (Li et al. 2019; Zhong et al. 2014). CRKP possessed potent resistance to 20 antimicrobials in the current 31 antimicrobial sensitivity assay (exceeding 90%), including an alarming 100% resistance to 17 antimicrobials, which was consistent with the results of researchers in Zhejiang Province, China (Zhan et al. 2017). This study suggests that β-lactam antibacterial drugs may no longer be effective against carbapenem-resistant *Klebsiella pneumoniae*, probably because the KPC enzyme was able to catabolize a variety of β-lactamases, including carbapenems, cephalosporins, penicillins, and aminotransim, which were some of the commonly used β-lactam antimicrobials in clinical practice (Queenan and Bush 2007). Whereas aminoglycosides might inhibit CRKP, and aminoglycoside-containing regimens might be an effective treatment option for the infections caused by CRKP strains, the situation is not promising because aminoglycoside-resistant genes are common in these strains (Huang et al. 2022). The antimicrobials of tetracycline and glycopeptide, on the other hand, showed strong inhibitory effects against CRKP, but a small number of CRKP strains in this study were still insensitive to the antimicrobials of tetracycline and glycopeptide and were able to withstand the pressure from the antimicrobials of tetracycline or glycopeptide, suggesting that CRKP was gradually becoming resistant to the antimicrobials of tetracycline and glycopeptide, and perhaps a combination of tetracycline and glycopeptide antimicrobials may be a treatment strategy for patients infected with CRKP resistant to the antimicrobials of tetracycline and/or glycopeptide (Tian et al. 2021).

The principle behind the MLST scheme is to use 7 housekeeping genes (internal nucleotide sequences of about 400 to 500 bases). Random integers are assigned to the unique sequences (alleles) of 7 housekeeping genes, and these random integers can combine into the unique alleles of each locus, the “allelic signature,” to obtain the multi-locus sequence type (ST). To this day, MLST is considered the “gold standard” for species typing (Aanensen and Spratt 2005). While in the USA and European countries, *K. pneumoniae* ST258 contributes significantly to the spread of carbapenem resistance, in the Chinese region, ST11 dominates; ST11 is a single motif variant (TonB) of ST258 and they are closely related. It was reported that ST11 and ST258 belonged to the clonal complex of CC258, which included ST11, ST258, and five other STSs (ST270, ST340, ST379, ST407, and ST418) (Qi et al. 2011; Wang et al. 2018). The CRKP isolated from this tertiary care hospital had a predominant ST11 type, and the number of ST11 type of CRKP in this tertiary care hospital showed a significant annual increase over the 5-year period from 2016 to 2020. This is consistent with the results of other Chinese researchers (Zhao et al. 2021; Hu et al. 2020). All CRKPs could be clearly divided into 5 taxa in the PFGE clustering analysis, and taxon A was full of ST15 clonal CRKP but had a low number of 3 strains compared to the results of a study in a medical center in northeastern China (Chen et al. 2021). CRKP of taxa B, C, D, and E were all dominated by ST11, but there were still some differences even among the same ST11 clonotype, which was probably because multiple incompatibilities between plasmids could lead to different resistance genes carried on the plasmids of each isolate, thus resulting in individual differences (Kim et al. 2021).

It was found in the virulence gene test results that virulence genes were more abundant in some CRKPs compared with the results of other researchers, especially the detection rate of *rmpA, magA*, and *iutA* genes was significantly higher than that of isolates from a tertiary hospital in Chongqing area (Zeng et al. 2021), but the results were closer to those of a tertiary care hospital in the Zhejiang area (Zhan et al. 2017). The *rmpA*, *magA*, *and iutA* virulence genes tend to be common in HVKP, which may be a sign that the CRKP within this hospital is evolving toward high virulence. The virulence plasmids which were considered non-conjugated in most previous studies are present only in HVKP. Now there has been a worldwide spread of virulence plasmids from HVKP into CRKP, in which the plasmids are increasingly associated with the spread of virulence factors, leading to the spread of important pathogenic characteristics, with CRKP possessing highly virulent plasmids being considerably more pathogenic than before (Kopotsa et al. 2020; Russo et al. 2014; Zhang et al. 2020b). Interestingly, a *bla*_NDM-1_ carbapenemase-type CRKP strain had capsular serotype K54, which was the first time in the region that a CRKP isolate was found to have capsular serotype K54; however, according to related reports, K54 was one of the supervirulent capsular serotypes of *K. pneumoniae* (Turton et al. 2018). The virulence gene combination of this *bla*_NDM-1_ isolate was WcaG-urea-wabG-fimH-entB-KfuB-alls-uge-ycf with two additional virulence genes of KfuB-alls compared with the virulence gene combinations of other isolates; the results of the correlation study concluded that there was a strong correlation between kfuB, alls, and K1 capsular serotype isolates, and that all K1 strains were considered positive for kfuB and alls (Yu et al. 2008). However, the podosome serotype K54 of the CRKP strain isolated in this study possessed the combination of KfuB-alls, which is a very special finding. The virulence plasmid expressing K54 serotype might be accidentally obtained during the transmission of this isolate, which meant that this isolate had both high resistance of ST11 and high virulence of K54. Subsequent studies may be conducted to verify the plasmid horizontal transfer ability of this strain and to obtain the complete genetic information of its plasmid to determine the cause of this phenomenon (Yang et al. 2021).

The resistance genotypes of CRKP collected during our study were mainly the prevalent genotype of *bla*KPC-2, with *bla*NDM-1 and *bla*NDM-5 resistance genotypes accounting for very few proportions. The types of outer membrane proteins were dominated by OmpK35 and OmpK37. The vast majority of isolates contained both OqxA and OqxB efflux pump genes. ST types were mainly ST11, with ST15, ST273, ST626, and ST340 accounting for a few proportions. The strains with *bla*KPC and *bla*NDM resistant genotypes showed very strong resistance to common antimicrobials, and there were also individuals with abundant virulence genes in these CRKPs, which means that some CRKP strains may gradually become novel *K. pneumoniae* with mixed characteristics of super-resistance and high virulence, so it is a very necessary task to timely monitor, prevent, and control CRKP epidemic disease in hospitals.

### Supplementary Information

Below is the link to the electronic supplementary material.

Supplementary file1 (XLSX 68 KB)

## Data Availability

The original contributions presented in the study are included in the article/Supplementary Material; further inquiries can be directed to the corresponding authors.
